# Mild reproductive impact of a Y chromosome deletion on a C57BL/6J substrain

**DOI:** 10.1007/s00335-017-9680-0

**Published:** 2017-03-10

**Authors:** Megan M. MacBride, Adam Navis, Amar Dasari, Ana V. Perez

**Affiliations:** Taconic Biosciences, One Hudson City Centre, Hudson, NY 12534 USA

## Abstract

A recently reported deletion of about 40 Mb in length between 6.12/6.57 and 46.73/47.31 Mb on the Y chromosome long arm of the C57BL/6JBomTac inbred strain made us closely examine the strain’s breeding history and reproductive characteristics. We verified that the two copies of *Rbm31y* that are present inside the putative deletion were indeed deleted. This inbred strain presents an expected litter size for a C57BL/6 substrain. In vitro fertilization (IVF) efficiency and breeding efficiencies are comparable to those of the C57BL/6NTac substrain; however, the male/female sex ratio in the C57BL/6JBomTac is mildly skewed towards females. There is an increase in the percentage of sperm shape abnormalities found in C57BL/6JBomTac (35%) versus C57BL/6NTac (11%). The most frequent type of sperm abnormality observed is bent heads (19%). Additionally, there is deregulation of several transcripts expressed in the testes. We determined that this mutation arose in the C57BL/6JBomTac Foundation Colony in 2008, and it was completely fixed in the colony by 2009.

**Electronic supplementary material:**

The online version of this article (doi:10.1007/s00335-017-9680-0) contains supplementary material, which is available to authorized users.

## Introduction

The Y chromosome in mammals is quite unique; it is the smallest of the chromosomes and is found only in males. In the mouse, the Pseudo Autosomal Region (PAR) is found at the tip of the Yq long arm and it spans about 700 Kb. This is the site of obligatory pairing and recombination between the X and Y chromosomes during male meiosis (Perry et al. 2001). The rest of the Yq chromosome is the non-pairing Yq region (NPYq) or male-specific Y chromosome (MSY) region that is thought to be involved in spermatogenesis and fertility (Burgoyne et al. 1992; Burgoyne 1998). The mouse MSY is quite large (89.6 Mb) when compared to human (22.8 Mb), chimpanzee (25.8 Mb), and rhesus (11 Mb) (Soh et al. 2014). The sequencing of the mouse MSY was an endeavor that took 12 years. It corroborated previous studies that showed several MSY genes present in multiple copies in the order of hundreds (Conway et al. 1994) and other studies that showed many of these genes to be amplified *en bloc* and transcribed bidirectionally (Ellis et al. 2007). Additionally, these studies showed that these genes are mostly expressed in the testes (Toure et al. 2005; Cocquet et al. 2009; Ellis et al. 2011). The mouse sequencing project provided the detailed architecture of the ampliconic 86.4 Mb of MSY sequence which consists of a 0.5 Mb unit amplified 200 times (Soh et al. 2014).

GVG Genetic Monitoring informed us of a 40 Mb deletion in the C57BL/6JBomTac inbred strain detected using short tandem repeat (STR) analysis (Fischer et al. 2016). This deletion represents 46% of the MSY. In this study, we verify a deletion in the Y chromosome of C57BL/6JBomTac mice, and we evaluate the effects of this deletion on the reproductive characteristics of this inbred strain. Another C57BL/6 substrain, C57BL/6NTac, was used as a comparator for many of the experiments described.

## Materials and methods

### Mouse strains

C57BL/6JBomTac (B6JBom) and C57BL/6NTac (B6NTac) male mice at 11–24 weeks old, and B6JBom and B6NTac female mice at 3–4 weeks old, all from Taconic Biosciences were used in all experiments. All applicable international, national, and/or institutional guidelines for the care and use of animals were followed.

### DNA and RNA isolation, cDNA synthesis, and qPCR

Mice were euthanized according to the protocols approved by the IACUC. The testes were removed and total RNA was extracted using the Qiagen RNeasy-Mini Kit (Qiagen, Valencia, CA) following the manufacturer’s protocol. Genomic DNA was extracted from tail biopsies using the Qiagen DNeasy Blood and Tissue Kit (Qiagen, Valencia, CA) following the manufacturer’s protocol.

For real-time reverse transcriptase polymerase chain reaction (RT PCR), total testis RNA was extracted and amplification of cDNA was performed using 1 µg of purified RNA with anchored oligo-dT primers (NEB, Ipswich, MA) using M-MuLV reverse transcriptase (NEB, Ipswich, MA), following the manufacturer’s recommendations.

List of primers used for genomic PCR amplification and gene expression is provided as Table S1 (Online Resource). Gene expression was analyzed using the 2^ΔΔCt^ method. All genes were normalized to *β-actin* and the expression level for each gene is presented as the ratio of B6JBom divided by B6NTac. The threshold for statistical significance was set at *p*-value of 0.05 and was generated using Microsoft Excel 2-tailed Student’s *t*-test.

### Mouse breeding performance and female/male sex ratio

Mice were bred in Taconic Isolated Barrier Units and were fed NIH #31M Rodent Diet. Breeding performance indicators such as production efficiency index (PEI), which is the measure of the total number of pups weaned per breeding female, and litter size were collected on a weekly basis. Female and male sex ratios were calculated by the proportion of males and females produced by each strain.

### Assessment of sperm morphology

The cauda epididymis from each male was dissected and placed into a dish containing a 120 µl drop of sperm freezing media (R183S) under oil at room temperature. The epididymis was then cut with fine scissors and gently pressed to release the sperm. Using fine forceps, the sperm clot was pulled into the freezing medium drop and allowed to disperse for a few minutes. An aliquot of sperm was mixed with Eosin-Nigrosin stain (Newcomer supply, Middleton, WI, USA) following the manufacturer’s recommendations, and smears were made on clean glass slides. The morphology of the sperm was assessed using light microscopy at 1000× magnification. Sperm morphology analysis was performed for at least three males from each strain, and a minimum 150 spermatozoa were analyzed per male. Sperm with any deviation from normal morphology was counted as abnormal. Sperm head abnormalities were classified as slight or gross abnormalities (Yamauchi et al. 2009). Microsoft Excel was used to calculate standard deviations and statistical significance set at *p*-value of 0.05 using 2-tailed Student’s *t*-test.

### In vitro fertilization

In vitro fertilization was performed following the protocol described by Takeo and Nakagata (2010). Briefly, pre-pubertal female mice were superovulated by intraperitoneal injections of 5 IU of equine chorionic gonadotropin (eCG), followed by human chorionic gonadotropin (hCGn) at 48 h interval. Oviducts were collected at 14–15 h post hCG injection, and transferred into a dish containing a 90 µl drop of FERTIUP fertilization medium (Cosmo Bio USA, Carlsbad, CA) under oil. Using a fine forceps, cumulus-oocyte complex (COC) was pulled from ampulla of oviducts into the fertilization drop and incubated at 37 °C for 30 min. Cauda epididymal sperm was placed into a 100 µl drop of FERTIUP sperm pre-incubation medium (Cosmo Bio USA, Carlsbad, CA), and incubated at 37 °C for 60 min. After 60 min, actively motile sperm from the periphery of drop was collected and added to the oocytes in the fertilization drop and co-incubated for 3 h. Then the oocytes were washed of excess sperm and cultured overnight for development to 2-cell embryos.

### Single-nucleotide polymorphism (SNP) analysis

Tail biopsies were sent to the University of North Carolina, Systems Genetics Core for GigaMuga SNP analysis (Morgan et al. 2016). 143,259 SNP probes were tested along the mouse genome, with 83 SNPs in the Y chromosome. SNP genotyping was done at Taconic’s Molecular Biology Laboratory using various SNP marker panels developed by Taconic, such as 96 SNP marker GenMon panel or 1449 SNP marker panel using Illumina’s platform (Illumina, Inc., San Diego, CA), following the manufacturer’s protocols. SNP analysis was done using Genome Studio Software (Illumina, Inc, San Diego, CA).

### Short tandem repeat (STR) analysis

Tail biopsies were sent to GVG Genetic Monitoring (Leipzig, Germany) to be tested by a panel of 246 STR markers along all mouse chromosomes, 18 of them on the Y chromosome. List of STR markers and their Y chromosome position used by GVG are provided on Table S2 (Online Resource).

### Multiparametric flow cytometry analysis on spleen and bone marrow

Mice were sent to Maine Medical Research Institute for analysis. Mice were euthanized and spleens were dissected onto a petri dish. Each spleen was minced using a scalpel and small scissors. Five milliliters of RPMI media were added to the minced tissue and then transferred into a syringe with a 70 µm filter. Cells that passed through the filter were collected into a 50 ml conical tube. The filter was washed with additional RPMI and cells were pooled with first pass-through. The cell suspension was spun at 500 g for 5 min. The supernatant was discarded and cells were resuspended in 10 ml of FACS buffer (PBS/0.5%BSA/2 mM EDTA). Cells were passed once more through a 70 µm filter. Cells were counted and diluted into a single cell suspension of 1 × 10^7^ cells/ml. Splenocytes were placed on ice until flow cytometry commenced.

For bone marrow isolation, hind legs of euthanized mice were removed and femurs and tibia were dissected from surrounding tissue using sterile scissors exposing the interior marrow shaft. The contents were flushed from the marrow with RPMI using a 5 ml syringe and a 27G × 1/2 needle and collected into a 50 ml conical tube and filtered through a 70 µm filter. Contents were spun at 500 g for 5 min, the supernatant was discarded and cells were resuspended in 10 ml of FACS buffer (PBS/0.5%BSA/2 mM EDTA). Cells were counted and diluted into a single cell suspension of 1 × 10^7^ cells/ml. Bone marrow cells were placed on ice until flow cytometry commenced. *P* values were calculated using unpaired *t* test.

Bone marrow and splenic cell suspensions were used to analyze subpopulations of myeloid cells and lymphocytes. The total number of viable cells was also determined. Cells were treated with TrueStain fcX (Biolegend, San Diego, CA) to prevent non-specific binding. Cells are then stained with antibodies against specific cell surface markers. Multiparametric flow cytometry analysis was performed using a combination of antibodies (see Table S3 Online Resource). DAPI was used to exclude dead cells. At least 10,000 events were collected using MACSQuant 10 analyzer (Miltenyi Biotec). Flow cytometric analysis was done using WinList 5.0. *P* values were calculated using unpaired *t* test.

## Results

### STR analysis shows absence of specific STR markers

**Fig. 1:**
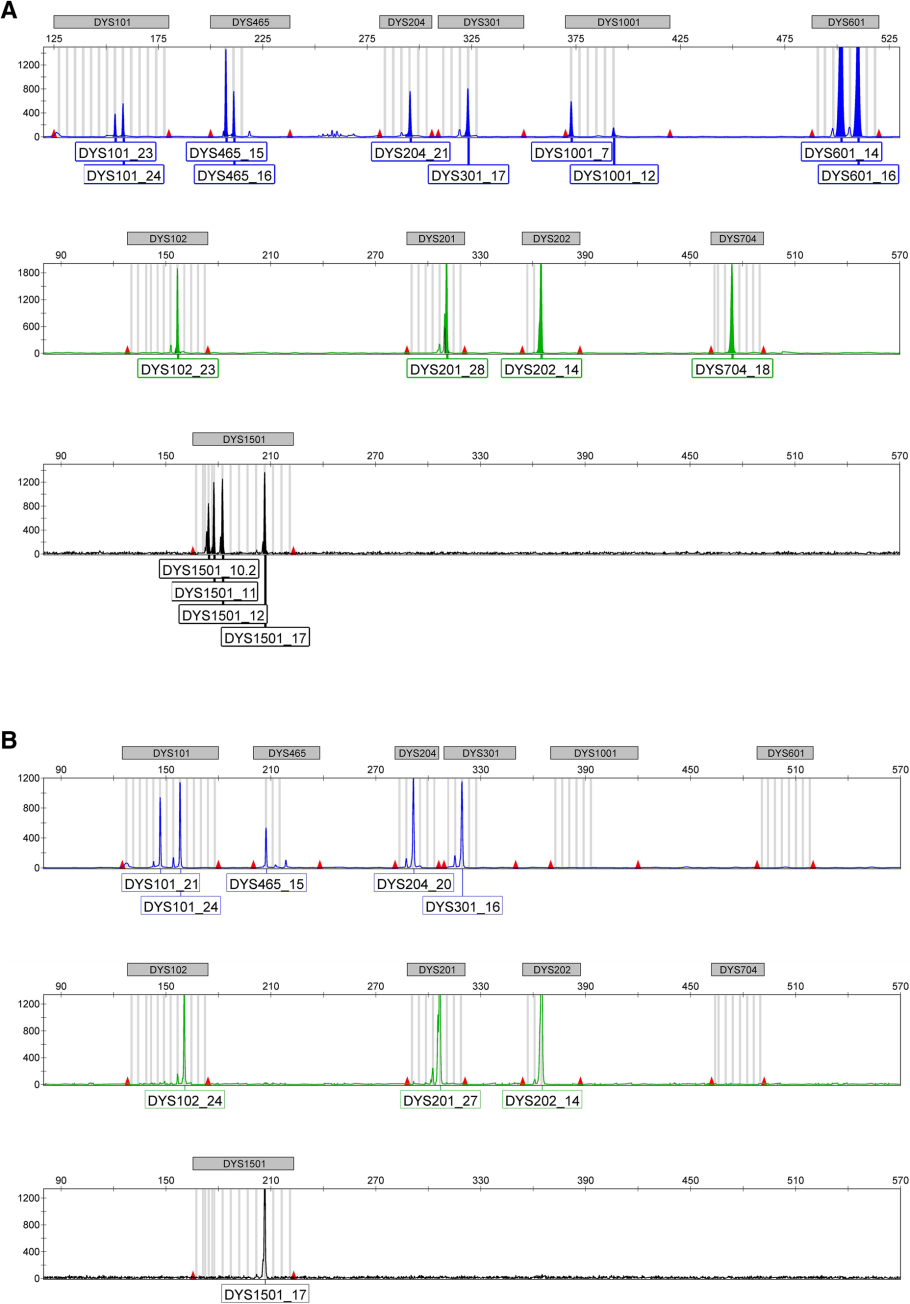
Electropherograms of multiplex STR profile of 11 Y chromosome STR markers in C57BL/6NTac and C57BL/6JBomTac. *Y-axis* represents the fluorescence intensity of the amplified band and the *X-axis* represents approximate size of the amplified band in base pairs. Since some primer pairs target more than one specific region, a total of 18 target regions are analyzed simultaneously. **a** C57BL/6NTac male Y chromosome STR profile shows all 18 STR markers. **b** C57BL/6JBomTac male Y chromosome STR profile shows only nine STR markers

Personal communication from GVG Genetic Monitoring had alerted us of a Y chromosome deletion of approximately 40 Mb on the B6JBom strain. In order to verify that B6JBom indeed lacked various STR markers in the Y chromosome, we sent samples from B6JBom and B6NTac females and males from our colony to GVG Genetic Monitoring. The data showed that, indeed, some markers localized between 6.57 and 46.73 Mb (personal communication by GVG, Fischer et al. 2016) were absent in the electropherograms from B6JBom shown on Fig. 1. B6NTac presented with the 18 expected microsatellite markers present (Fig. 1a), while only 9 of the 18 markers were detected in B6JBom (Fig. 1b). Markers absent are between position 12.87 and 46.6 Mb (Table S2). The size and location of the mutation between 6.12/6.57 and 46.73/47.31 Mb have been defined by GVG (Fischer et al. 2016).

### GigaMuga SNP analysis does not provide informative SNPs in the Y chromosome in the region of the putative deletion

In order to verify the reported Y chromosome deletion with another method, tail biopsies of B6JBom and B6NTac mice were sent to the University of North Carolina Systems Genetics Core to be tested with the GigaMuga SNP Panel (Morgan et al. 2016), which has 83 SNP markers in the Y chromosome. No unique SNP markers were found in the region of the putative 40 Mb deletion, and we were not able to verify the presence of the deletion with this methodology (data not shown).

### *Rbm31y* is deleted in B6JBom

**Fig. 2:**
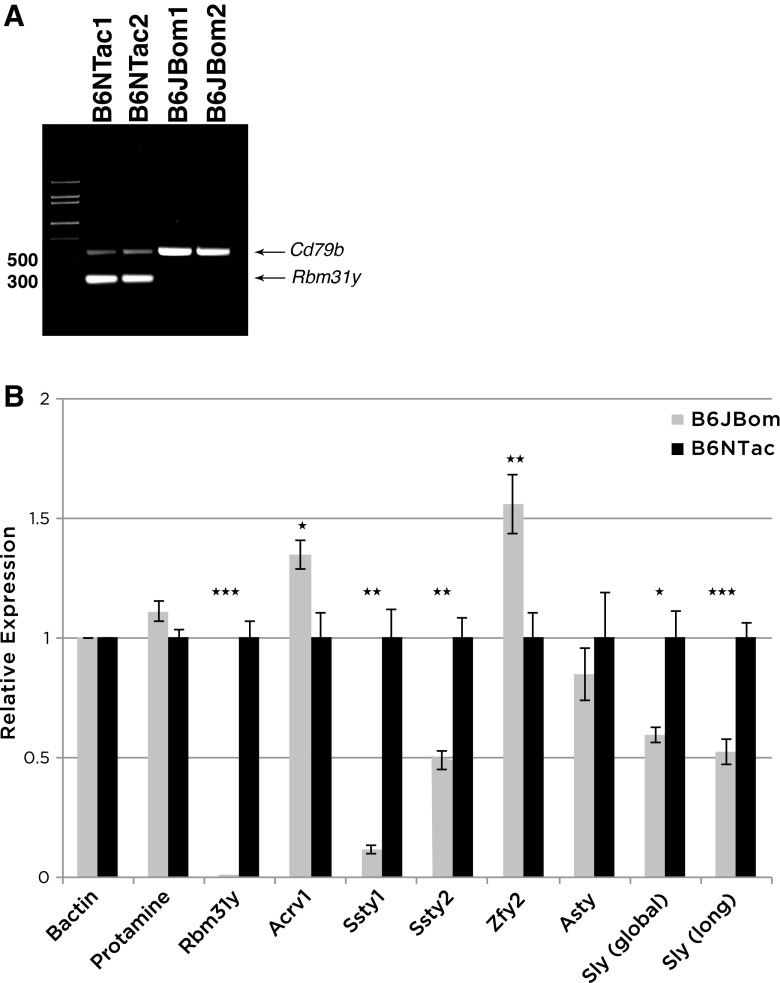
Genomic DNA PCR amplification of *Rbm31y* and transcript level of *Rbm31y* and other Y chromosome genes. **a** PCR amplification of genomic DNA from B6NTac and B6JBom males using primers for a housekeeping gene *Cd79b* and Y chromosome encoded *Rbm31y*. **b** Expression level of selected Yq transcripts: *Rbm31y, Ssty1, Ssty2, Asty, Sly* global (*Sly1* and *Sly2*), and *Sly* long (only *Sly1*), a Yp gene: *Zfy2*, and selected genes encoded on other chromosomes: *Prm1* (Chromosome 16); *Acrv1* (Chromosome 9), and housekeeping gene *β-actin* (Chromosome 5) from male B6NTac and B6JBom. *P* values are represented by asterisks; *p < 0.05, **p < 0.01, ***< *p* < 0.001

We extracted from the NCBI database all reported genes and open reading frames within the putative deleted region (Online Resource Table S4) and found that gene *Rbm31y* has two copies in the putative deleted area and none outside of this region. We designed primers based on sequence from the NCBI database. PCR genotyping results show that *Rbm31y* is indeed deleted (Fig. 2a).

### Genes on both the Y chromosome and the autosomes are deregulated

Mice with Y chromosome deletions were previously reported to have deregulation of Y chromosome genes expressed in the testes (Reynard and Turner 2009; Ellis et al. 2005). We therefore quantified levels of selected transcripts in testes from B6JBom and B6NTac. As expected, *Rbm31y* transcripts were missing in B6JBom since the 2 copies of this gene are deleted (Fig. 2a, b). The levels of *Acrv1* and *Zfy2* transcripts were increased while the levels of *Ssty1, Ssty2*, and *Sly* global (*Sly1* and *Sly2*) and *Sly* (*Sly1* only) transcripts were significantly decreased in B6JBom compared to B6NTac.

### B6JBom males have normal breeding performance but distorted male/female sex ratio

The PEI was collected from Taconic Murine Pathogen-Free^™^ (MPF^™^) colonies containing 1:1 breeding pairs. There was no difference between the PEIs of B6JBom and B6NTac (Table 1).

**Table 1:** B6JBom and B6NTac breeding performance

Strain	Health Standard	PEI
C57BL/6NTac	MPF	0.76
C57BL/6JBomTac	MPF	0.74

A bias towards females in the male to female sex ratio has been previously reported for Y chromosome deletion mutants (Styrna et al. 1991; Conway et al. 1994; Ward and Burgoyne 2006). We reviewed historical information on both Taconic B6 substrains to see if there was such a bias in the B6JBom substrain. We found that in 2009, there was a shift in the B6JBom male/female sex ratio, and the shift has been maintained since then in our breeding colonies (Table 2). The B6NTac substrain male/female sex ratio remained ~1.00 during the same period.

**Table 2:** B6JBom and B6NTac male/female sex ratio

M/F sex ratio		
Year	C57BL/6JBomTac	C57BL/6NTac
2004	1.12	1.07
2005	1.1	1.04
2006	1.1	1.04
2007	1.09	0.94
2008	0.99	0.9
2009	0.76	0.99
2010	0.83	1.01
2011	0.77	1.02
2012	0.79	1.00
2013	0.8	1.03
2014	0.87	1.04
2015	0.77	1.00
2016	0.82	1.03

### Fertilization rate of B6JBom is comparable to B6NTac

In order to assess whether the Y chromosome deletion had any impact on IVF rates, we did a comparative study using sperm from B6JBom and B6NTac. Two sets of IVF experiments were performed, the first with sperm from four individual males and the second with three individual males, each male tested with two sets of pooled oocytes from five females for B6JBom. For B6NTac, five individuals males were used each tested with two sets of pooled oocytes from five females. No differences in the IVF fertilization rates, measured as a proportion of inseminated oocytes that developed to the two-cell stage embryos, were observed (Table 3).

**Table 3:** B6JBom and B6NTac in vitro fertilization rates

Strain	# Female donors	% Fertilization rate*
C57BL/6JBomTac	70	85 ± 6
C57BL/6NTac	50	88 ± 7

*Average ± SDev

### Sperm morphology analysis shows a range of sperm abnormalities in B6JBom

**Fig. 3:**
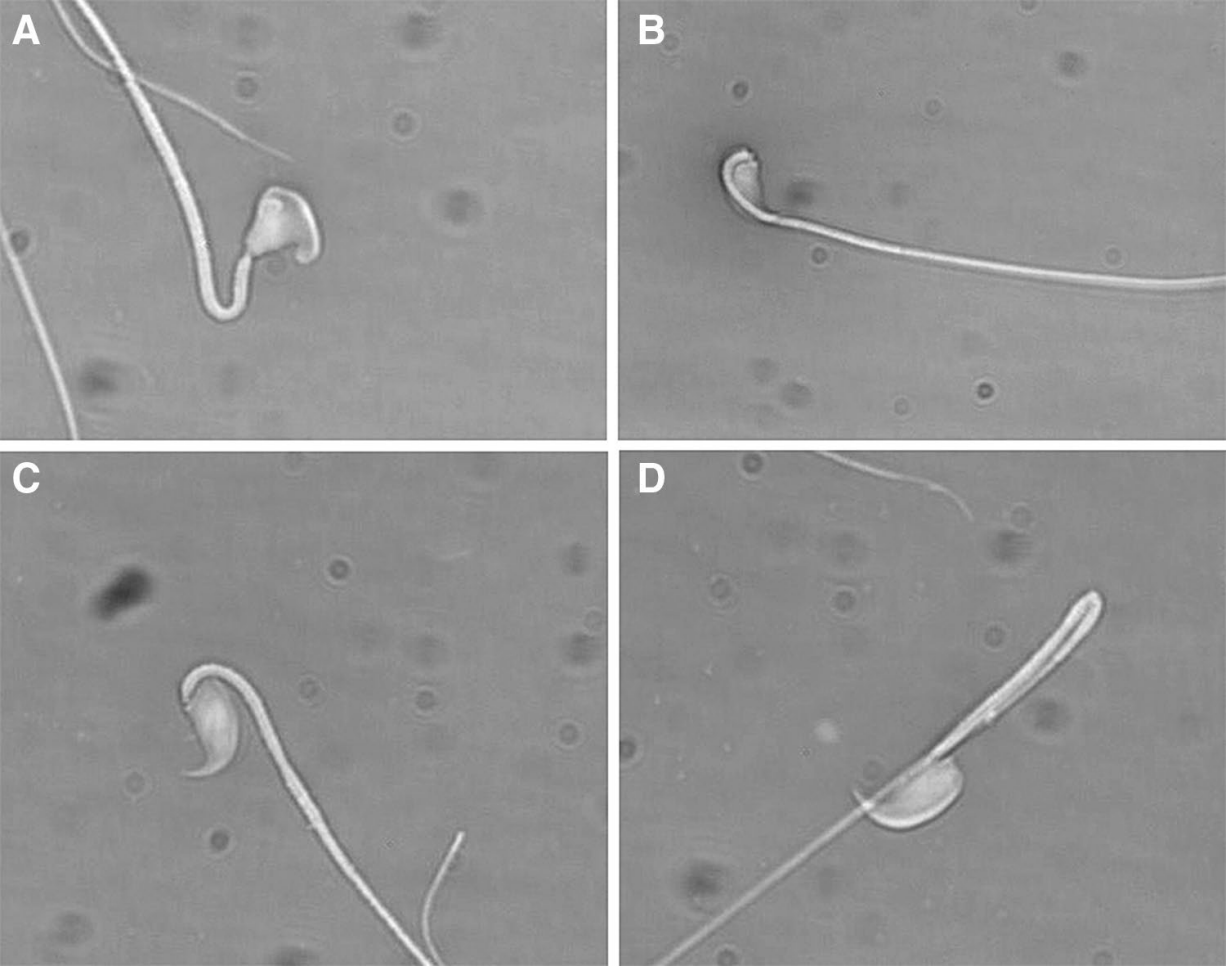
Sperm shape abnormalities observed in B6JBom males. **a** Abnormal head; **b** and **c** bent head; **d** normal head and bent tail. Magnification is 1000×

Abnormal sperm morphology has been previously observed in various Y chromosome deletion mutants and the severity of the deletion correlates with the percentage of abnormal sperm (Styrna et al 1991, Toure et al 2004, Ward et al 2006). We quantified the number of sperm with normal and abnormal shape, and among abnormal sperm differentiated between those with bent heads and those with abnormal head shape. B6JBom males had significantly more morphologically abnormal sperm (35 vs. 11%, *P* < 0.05) than B6NTac (Table 4). The predominant defect observed in B6JBom was ‘bent heads’ (Table 4; Fig. 3b–c).

**Table 4:** B6JBom and B6NTac sperm morphology defects

Strain	% Abnormal sperm	% Bent heads	% Abnormal head
C57BL/6JBomTac	35 ± 1*	32 ± 2*	3 ± 1
C57BL/6NTac	11 ± 5*	2 ± 0*	8 ± 5

*P-value < 0.05

### Some statistically significant differences in immune cell frequency or count, but no immunodeficiency

**Fig. 4:**
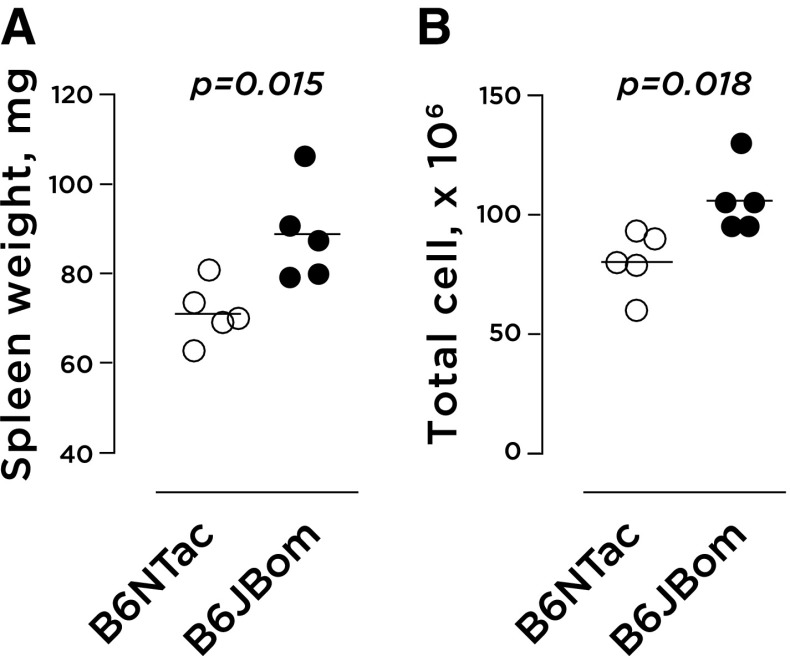
Spleen size in B6JBom and B6NTac males. **a** Spleen weight; **b** spleen cell number

**Fig. 5:**
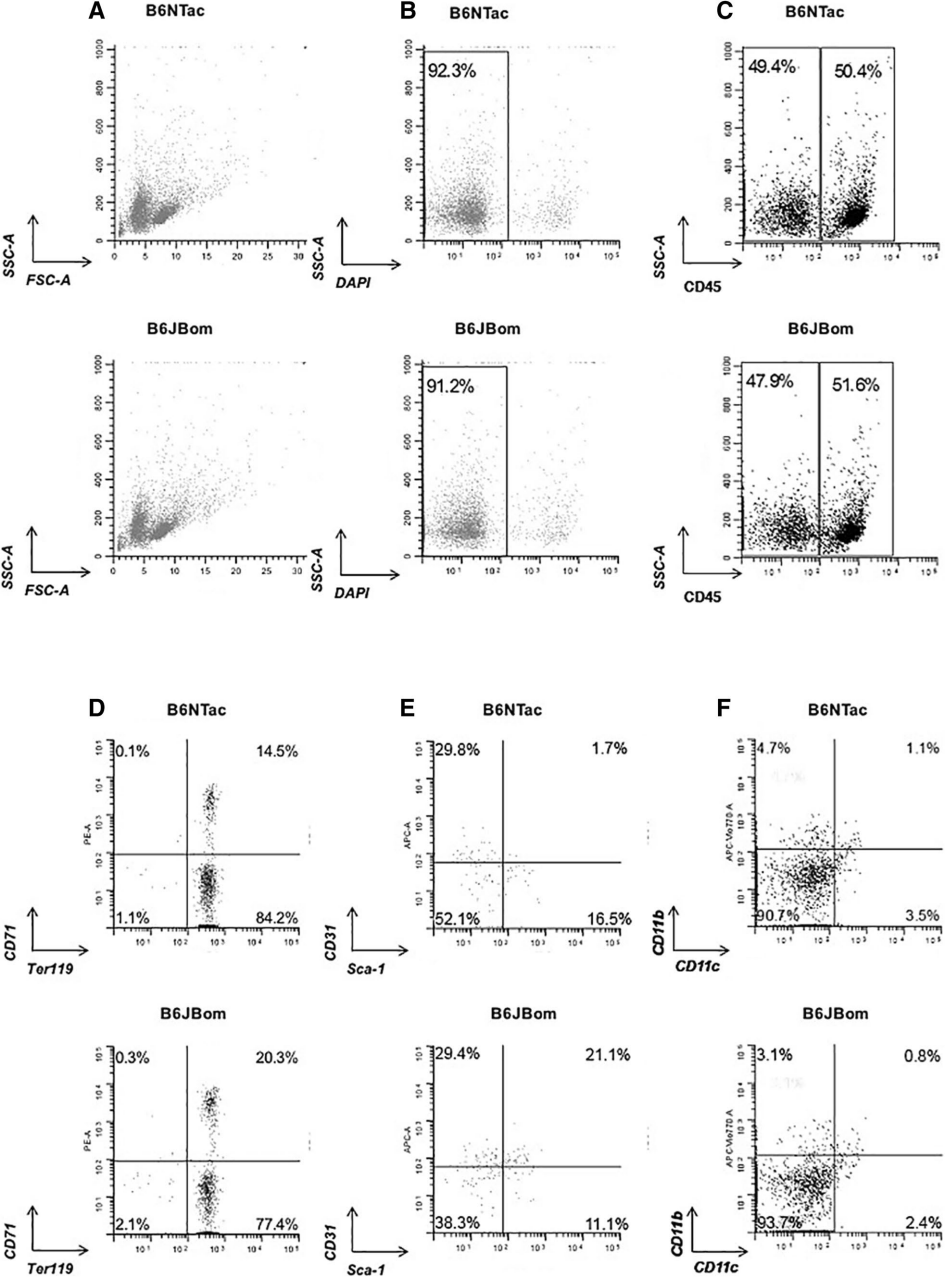
Flow cytometry characterization of mouse spleen cells from B6NTac and B6JBom males. **a** SSC/FSC characteristics of spleen cell suspension; **b** viable cells from spleen cell suspension were stained with DAPI. A total of 50,000 events were collected. **c** Viable CD45−, non-immune cells (*left* gate) versus CD45+, immune cells (*right* gate). **d** Viable erythrocytes (CD45−, CD71−, Ter119+) versus mature erythrocytes (CD45−, CD71+, Ter119+). **e** Viable endothelial cells (CD45−, CD71−, Ter119−, CD31+) versus fibroblasts (CD45−,CD71−, Ter119−, Sca-1+). **f** Viable myeloid cells (CD45+, CD11b+) versus dendritic cells (CD11c+, CD11b−). *Top row* B6NTac, *bottom row* B6JBom

A recent publication (Sun et al. 2013) presented evidence of a potential link between Y chromosome deletion and B and NK cell deficiencies. To investigate potential differences in the immune characteristics between B6JBom and B6NTac, a multiparametric flow cytometry analysis of spleen and bone marrow cells was performed. B6JBom presented with larger spleens when compared to B6NTac, evidenced by a higher organ weight and a higher number of spleen cells (Fig. 4). The distribution of all cells (Fig. 5a), viable cells (Fig. 5b) and immune (CD45+) and non-immune (CD45−) cells (Fig. 5c) was similar in spleens from B6JBom and B6NTac. However, in-depth characterization of CD45− and CD45+ spleen cell subpopulations revealed some differences between B6JBom and B6NTac (Fig. 5d–f, S2, Table S5). B6JBom had more mature erythrocytes but fewer T lymphocytes, T helper cells and cytotoxic T cells, natural killer cells, myeloid phagocytic cells, and macrophages, when compared to B6NTac.

The analysis of immune and non-immune bone marrow cell populations did not show any significant differences between the B6 substrains (Table S6). There were also no differences observed in immune and non-immune cell populations from spleen or bone marrow of female B6JBom and B6NTac mice (data not shown).

## Discussion

All the evidence acquired in this study, including absence of both copies of the *Rbm31y* gene, altered levels of examined transcripts in testes, biased male/female sex ratio, and sperm shape abnormalities, supports the presence of a fixed Y chromosome deletion in Taconic’s B6JBom inbred strain, reported recently by Fischer et al. 2016. With respect to reproductive abilities, this deletion thus far seems to mostly affect sperm morphology, with a mild impact on the male/female sex ratio, but no alterations on the natural mating breeding performance or IVF efficiency. The data indicate that for the most part, the phenotype observed mainly affects the male germline, which is in agreement with several publications that describe other mouse and human Y chromosome deletions (Conway et al. 1994; Reynard and Turner 2009; Toure et al. 2004; Ma et al. 2000).

In mice, the Y chromosome long arm (Yq) consists almost entirely of massively amplified sequence, with three primary gene families, *Sly, Sry*, and *Ssty*. These genes are present in 132, 197, and 317 copies, respectively (Soh et al. 2014). This highly repetitive sequence has made interrogation of the mouse Y chromosome difficult.

Our goal was to confirm the reported mutation and understand the phenotypic impact of this deletion to the widely used B6JBom inbred strain. Our study provided additional supportive evidence to a recently reported 40 Mb deletion that has been mapped close to 6.12/6.57 and 46.73/47.31 Mb in the Y chromosome of B6JBom (Fischer et al. 2016) and that has become fixed in the Taconic B6JBom foundation colony. The STR marker analysis showed the absence of markers in the Y chromosome of B6JBom, and we verified that both copies of *Rbm31y* were deleted from the B6JBom genome. However, the high density SNP panels, the current gold standard for mouse genetic monitoring, were uninformative due to a lack of unique SNP markers in the mouse Yq arm.

We observed a specific testicular phenotype in B6JBom males as compared to B6NTac controls. Specifically, we observed alterations in levels of testis transcripts of several genes. Similar transcriptional alterations were observed before in mice with known NPYq deletions, and were shown to correlate with the deletion size (Toure et al. 2005; Ellis et al. 2005; Reynard and Turner 2009; Coquet et al. 2009). The reduced transcript levels of genes encoded within NPYq of B6JBom (*Sly, Ssty*, and *Asty*) are reflective of the loss of several of their copies encoded within the deleted region. Our reported B6JBom deletion is the smallest of all reported thus far, approximately 40 Mb, equivalent to a 46% deletion of the Y chromosome, representing a 40% reduction on the levels of *Sly* transcription. The smallest previously reported Yq deletion mutant, Del(Y)B10.BR-Y^del^/Ms, which had 66% of Y chromosome deleted, presented with a 46% reduction on *Sly* transcription (Toure et al. 2005) which is a higher reduction than in B6JBom.

A similar pattern was observed when the expression of *Ssty1* and *Ssty2* in B6JBom was related to that reported for Del(Y)B10.BR-Y^del^/Ms. For all previously reported models with NPYq deletions, the exact deletion coordinates have not been determined. It is therefore possible that the differences in transcript expression between our study and those of others are due not only to differences in deletion size but also its location. It would be interesting to determine more precisely the gene breakpoints of these other NPYq deletion mutants.

NPYq encoded *Sly* has been shown to be a key regulator of sex chromosome gene expression by Coquet et al. (2009). Its deficiency or absence leads to a global upregulation of sex chromatin and occasional alterations of genes encoded on the autosomes. In agreement with the previous data by Cocquet et al. (2009), we demonstrated a significant upregulation of NPYp encoded *Zfy2* and autosomally encoded *Acrv1*, but lack of deregulation of autosomally encoded *Prm1. Zfy2* has been proposed to be essential in sperm head remodeling and sperm tail development (Vernet et al. 2016; Yamauchi et al. 2015), therefore upregulation of *Zfy*2 might explain the sperm abnormalities we see in B6JBom.

Other characteristics of MSYq deletion mutants are that they present sperm abnormalities, decreased rates of fertilization, and distortion of the male/female sex ratio (Xian et al. 1992). We did not observe differences in the reproductive performance or IVF rates between the B6JBom substrain compared to B6NTac. The PEI for B6JBom in Taconic’s MPF barriers is comparatively similar to B6NTac. Litter sizes for B6JBom are also as expected for a B6 substrain (6–8 pups/litter, data not shown). This lack of breeding deficiency is possible considering the size of deletion, the smallest of reported thus far.

In respect to sperm morphology anomalies, we observed 35% of sperm from B6JBom were abnormal, compared to 11% in B6NTac. We defined the severity of head deformities based on criteria described before (Yamauchi et al. 2009) and noted that most of head shape defects fell into the 1 and 2 S category (slight) with a few G (gross deformities). The observed sperm shape abnormalities did not negatively influence breeding or production efficiencies in breeding colonies. So while the mutation does cause alterations in sperm morphology, there is no impact on natural or assisted reproduction in B6JBom.

The male:female sex ratio distortion towards females observed in B6JBom due to the Y deletion is in agreement with skewed sex ratios reported for other mouse models with NPYq deletions (Styrna et al. 1991; Conway et al. 1994; Ward and Burgoyne 2006). This sex ratio bias has been hypothesized to reflect an intragenomic conflict between the X and Y chromosome (Coquet et al. 2009; Ellis et al. 2011; Coquet at al. 2012). Spermatid-specific multicopy Y chromosome transcripts, i.e., *Sly, Asty, Ssty1, Ssty2*, have a high degree of homology to multicopy spermatid-specific transcripts in the X chromosome, i.e., *Slx* and *Slxl1, Astx, Ssxb*. In mice with NPYq deletions, when the Y chromosome transcripts are downregulated, their X homologous counterparts are upregulated (Toure et al. 2005; Ellis et al. 2005; Raynard and; Turner 2009; Coquet et al. 2009).

The Y-encoded *Sly* and X-encoded *Slx*/*Slxl1* have been proposed to be the key players in intragenomic conflict in the mouse (Cocquet et al. 2009, 2012). *Sly* represses sex chromatin while *Slx*/*Slxl1* plays the opposite role and stimulates XY gene expression in spermatids. *Sly* deficiency leads to XY gene upregulation, sperm differentiation defects, subfertility/sterility, and sex ratio bias towards females (Cocquet et al. 2009). *Slx*/*Slxl1* deficiency rescues sperm differentiation defects and subfertility/sterility caused by *Sly* deficiency. Importantly, while *Sly* deficiency leads to sex ratio bias towards females, *Slx*/*Slxl1* deficiency causes a sex ratio distortion towards males (Coquet et al. 2012). These data support that *Sly* and *Slx*/*Slx1l* have antagonistic effects during sperm differentiation.

To investigate the origin of the deletion in our colonies, we looked into the breeding history of the B6JBom. The origin of Taconic’s C57BL/6JBomTac is as follows. The Jackson Laboratory received C57BL/6 in 1948 from Hall. In 1971, the Zentralinstitut fur Versuchsteirzucht in Hannover, Germany, received stock from the Jackson Laboratory. In 1988, M&B (now Taconic Biosciences) received stock from the Zentralinstitut fur Versuchsteirzucht. The mice were derived by Taconic in 2000 by embryo transfer. Taconic maintains the B6JBom as an inbred strain with a single Foundation Colony, which feeds all production colonies globally. Taconic’s Foundation Colony was refreshed in 2015 using cryopreserved embryos from 2010. We went back to 2004 records when reviewing the breeding history of this inbred strain. When we analyzed the collected breeding information from B6JBom and compared it to B6NTac, there was a clear breakpoint in the male/female sex ratio from 2008 to 2009. The male/female sex ratio shifted in the production colonies from a 1 to a 0.76 in 2009, and since then it has fluctuated between 0.76 and 0.82 (Table 2). This indicates that the mutation arose in the Foundation Colony, propagated to all production colonies and was fixed by 2009.

It is important to point out that the history of C57BL/6J (B6J), C57BL/6NTac, and C57BL/6JBomTac are of three different substrains that have independently drifted from each other. Jackson cryopreserved its B6J stock in 2003 at generation F226 to maintain their Genetic Stability program. The C57BL/6N (B6N) substrain diverged from B6J when stock was transferred from Jax to NIH in 1951. B6N was then transferred to Taconic from NIH at generation F151 in 1991. At this point, Taconic cryopreserved its B6NTac. Meanwhile, Jackson transferred B6J to Hannover in 1971, and in 1988 M&B (now Taconic Biosciences) received B6JBom from Hannover at F141, Taconic cryopreserved the B6JBom in 2010 at F207. Therefore, all 3 substrains are quite different and have drifted apart for at least 39 years (B6JBom from B6J), 40 years B6NTac from B6J (became N when it was received by NIH). We thus chose the B6NTac as a control for these experiments as a readily available strain that was separated from B6JBom a similar length of time as other possible control substrains.

Sun et al. (2013) reported a B and NK cell deficiency in the bone marrow of mice which was linked to a Y chromosome deletion of 1/3 of its length but were not able to determine how the Y chromosome deletion mediated B and NK cell defects. Here, we have shown differences between B6JBom and B6NTac in respect to spleen size and characteristics of immune and non-immune spleen cells. The biological relevance of these differences is not clear, but they do not seem to represent any sort of obvious immunodeficiency. These differences could be due to genetic modifiers characteristic of the B6JBom relative to the B6NTac substrain.

The finding of a Y chromosome deletion in the B6JBom strain was a surprise. From the perspective of animal production breeding, monitoring the Y chromosome has indeed been a challenge due to the nature of its many repeats and therefore lack of accessibility of its sequence. In the specific case of B6JBom, the genetic monitoring program was not able to detect the Y chromosome mutation due to the lack of informative SNP markers as previously discussed. While the B6JBom was cryopreserved per the requirements of the genetic quality program, the frozen stock already had the fixed Y chromosome deletion. We are working on developing additional markers in the repetitive region of the Y chromosome to include in our routine genetic monitoring program for all our inbred strains and outbred stocks. Taconic has a robust genetic quality program that aligns with industry standards. Inbred strains are cryopreserved and Foundation Colonies are refreshed every 5 years from cryopreserved stock to lessen the risk of genetic drift. All strains are tested using well characterized SNP panels performed on the Illumina platform. Breeding paradigms for inbred strains include unidirectional flow of mice, brother by sister mating as well as careful record-keeping. Breeding is harmonized globally for all production sites and distributors. In addition, we are in the process of performing whole genome sequencing of all our inbred strains.

Our study emphasizes on the importance of substrain differences. The Y chromosome deletion identified in B6JBom is not the first mutation that has been described on a C57BL/6 substrain; there are many known, and each of them present different effects in various organs/systems. The *Nnt* ^*C57BL*/*6J*^ mutation, a 17 Kb mutation that was discovered in B6J and affects several organ systems under stress conditions, has well-characterized effects on diabetes (Freeman et al. 2006). The *Snca* and *Mmrn1* deletions, a 300 Kb deletion discovered on the C57BL/6JOlaHsd (B6JOlaHsd), affect metabolism of brain chemical compounds (Specht and Schoepfer 2001; Gajovic et al. 2006). The *Crb1* ^*rd8*^ mutation, a one base-pair deletion in all commercially available B6N substrains, causes mild retinal degeneration which produces visual impairment but does not render the mouse completely blind (Mattapallil et al. 2012). The *Cyfip2* ^*Min*^ mutation, a G to A base change on all commercially available B6N substrains, causes acute and sensitized cocaine-response phenotypes (Kumar et al. 2013). As can be appreciated, findings of additional mutations in inbred strains will become more common as more substrains are sequenced. Research scientists should carefully choose a substrain when designing experiments and be cautious to be consistent in substrain usage for ongoing experiments.

## Electronic supplementary material

Below is the link to the electronic supplementary material.

Supplementary material 1 (PDF 744 KB)
