# Supplementary material for: Mild reproductive impact of a Y chromosome deletion on a C57BL/6J substrain
Source: Mamm Genome. 2017 Mar 10;28(5):155–65. doi: 10.1007/s00335-017-9680-0 (PMC5442250; doi:10.1007/s00335-017-9680-0)
Supplement: Supplementary file 1 — Supplementary material 1 (PDF 744 KB) [file 335_2017_9680_MOESM1_ESM.pdf]

Mild reproductive impact of a Y chromosome deletion on a C57BL/6J substrain

Megan M. MacBride, Adam Navis, Amar Dasari, and Ana V. Perez*
Taconic Biosciences, One Hudson City Centre, Hudson, NY 12534, USA

*To whom correspondence should be addressed (email: ana.perez@taconic.com, phone: +1 518-697-3984 fax: +1 518-697-3910).

Mammalian Genome

Table S1. List of Primers used on Testes Expression Analysis

Gene Name	Chrom #	Forward	Reverse	Reverse 2
Acrv1	9	TGAGTACACCACTTCCAAGCA	AAGCACATGTGTGGCAATTT	
Asty	Y	GRGGAGTAGAACTCATCATC	CAGGAGATGACTAACATAGCA	
ß-actin	5	GGCACCACACCTTCTACAATG	GTGGTGGTGAAGCTGTAGCC	
Protamine 1	16	ACAAAATTCCACCTGCTCACA	GTTTTTCATCGGaCGGTGGC	
Rbm31y Genomic	Y	TTTGGGATCGGCCAATATGC	ACTTCCTCCTCTTCCAACTGTC	
Rbm31y Expression	Y	AACCCAAATGCCTTCGAGTC	ACTTCCTCCTCTTCCAACTGTC	
Sly Global (*Sly1*+*Sly2*)	Y	CATTTATAAGACGCTTCACATAAAG	TCCTCCATGATGGCTCTTTC	ATTCTCCATGATGGCTCTTTC
Sly Long (*Sly1* only)	Y	GAAGACATGGGACATGAAGTAGG	TCCTCCATGATGGCTCTTTC	ATTCTCCATGATGGCTCTTTC
SSty1	Y	AGAAGGATCCAGCTCTCTATGCT	CCAGTTACCAATCAACACATCAC	
SSty2	Y	CAGGTGCCATTCTTACAGGACTAT	ACCCAGGAACCTATTAAGAAGTCAT	
Zfy2	Y	CTTAATTCCAGACATTTTAACTTCCA	ATCACTTGTTCAAAATGTCCTACATT	

Note: The primers '*Sly* global' amplify the two *Sly* transcripts (*Sly1* and *Sly2*), while the primers '*Sly* long' amplify only *Sly1*.

Table S2. List of STR Markers and their Y Chromosome positions

STR Marker #	Position Mb	Present in B6NTac	Present in B6JBom
DYS101	0.19	+	+
DYS101I	0.2	+	+
DYS102	0.33	+	+
DYS201	0.94	+	+
DYS202	1.73	+	+
DYS204	2.57	+	+
DYS301	3.99	+	+
DYS601	12.87	+	-
DYS704	17.38	+	-
DYS601I	17.58	+	-
DYS1001I	18.38	+	-
DYS1501I	35.69	+	-
DYS1501II	36	+	-
DYS1001	41.07	+	-
DYS465I	46.6	+	-
DYS465	46.7	+	+
DYS1501	81.16	+	+

Table S3. Antibodies used for multiparamentric flow cytometry analysis of spleen and bone marrow cells

Tissue	Antigen combinations	Target cell populations
Bone Marrow:<br>CD11b^{FITC},<br>CD19^{PE},<br>Ly6G^{PeCy7}<br>F4/80^{PerCP},<br>Ly6C^{APC},<br>CD45$^{APC/Cy7}$<br>DAPI	CD11b, Ly6G, Ly6C, F4/80	• Total CD11b^{pos}CD19^{neg}CD45^{pos} myeloid cell,<br>• CD11bLy6G^{pos} – granulocytic population,<br>• CD11bLy6C^{pos}Ly6G^{neg} – monocytic lineage,<br>• CD11bF4/80^{pos}/Ly6C^{neg} – BM macrophages & APC
	CD19, CD11b, CD45	• Total CD19^{pos} B lymphocytes,<br>• SSC^{int}/CD19^{pos}/CD45^{pos} – plasma B cells,<br>• SSC^{int}/CD19^{pos}/CD45^{neg} – abnormal B cells
	CD45^{pos}Lin^{neg}	• Total hematopoietic stem/progenitor cell (HPC)
Spleen:<br>CD11b^{FITC},	CD19, CD11b, CD45	• Total CD19^{pos} B lymphocytes
	CD3, CD8, CD4	• Total CD3^{pos} T lymphocytes;<br>• CD3^{pos}CD4^{pos} – Th cells<br>• CD3^{pos}CD8^{pos} – cytotoxic T cells

CD19PE, CD8PeCy7 CD11cPerCP, CD4APC, CD3APC/Cy7 DAPI	CD11b, CD11c, CD3, CD19	• CD11c^{pos}CD11b^{int}[CD3/CD19]^{neg} – migratory dendritic cells;<br>• CD11c^{pos}CD11b^{neg}[CD3neg/CD19]^{neg} – resident dendritic cells

Table S4. NCBI Database Extract of Y chromosome genes between 6.12 Mb and 47.31 Mb

Mouse Gene	Coding Region Position	Description	UCSC link
Rbm31y	chrY:17,400,788-17,402,485	RNA binding motif 31, Y-linked (Rbm31y), mRNA	http://genome.ucsc.edu/cgi-bin/hgGene?hgg_gene=uc029xsd.1&hgg_prot=Q9D3U4&hgg_chrom=chrY&hgg_start=17400760&hgg_end=17402718&hgg_type=knownGene&db=mm10&hgsid=500967801_Vakd6YZAzH49xqeBY7x8QEfmp51u
Rbm31y	chrY:12,688,137-12,689,834	RNA binding motif 31, Y-linked (Rbm31y), mRNA	http://genome.ucsc.edu/cgi-bin/hgGene?hgg_gene=uc012hsj.1&hgg_prot=Q9D3U4&hgg_chrom=chrY&hgg_start=12688109&hgg_end=12690067&hgg_type=knownGene&db=mm10&hgsid=500967801_Vakd6YZAzH49xqeBY7x8QEfmp51u
Sly	chrY:38,775,333-38,799,352	SAM and SH3 domain-containing protein 3	http://genome.ucsc.edu/cgi-bin/hgGene?hgg_gene=uc029xxu.1&hgg_prot=A0A087WSR0&hgg_chrom=chrY&hgg_start=38774735&hgg_end=38800793&hgg_type=knownGene&db=mm10&hgsid=500967801_Vakd6YZAzH49xqeBY7x8QEfmp51u
Ssty1	chrY:18,090,734-18,091,432	spermiogenesis specific transcript on the Y 1 (Ssty1), mRNA	http://genome.ucsc.edu/cgi-bin/hgGene?hgg_gene=uc009vif.2&hgg_prot=J3QPX2&hgg_chrom=chrY&hgg_start=18089166&hgg_end=21097715&hgg_type=knownGene&db=mm10&hgsid=500967801_Vakd6YZAzH49xqeBY7x8QEfmp51u
Ssty1	chrY:13,377,980-13,378,678	spermiogenesis specific transcript on the Y 1 (Ssty1), mRNA	http://genome.ucsc.edu/cgi-bin/hgGene?hgg_gene=uc029xsa.1&hgg_prot=J3QPX2&hgg_chrom=chrY&hgg_start=13376412&hgg_end=13378797&hgg_type=knownGene&db=mm10&hgsid=500967801_Vakd6YZAzH49xqeBY7x8QEfmp51u
Ssty2	chrY:22,513,860-22,514,543	spermiogenesis specific transcript on the Y 2 (Ssty2), mRNA	http://genome.ucsc.edu/cgi-bin/hgGene?hgg_gene=uc029xso.1&hgg_prot=Q149W4&hgg_chrom=chrY&hgg_start=22512350&hgg_end=22514683&hgg_type=knownGene&db=mm10&hgsid=500967801_Vakd6YZAzH49xqeBY7x8QEfmp51u

AB335742	chrY:43,265,812-43,265,855	non-coding RNA, oocyte_clustered_small_RNA943, complete sequence	http://genome.ucsc.edu/cgi-bin/hgGene?hgg_gene=uc029xye.1&hgg_prot=&hgg_chrom=chrY&hgg_start=43265811&hgg_end=43265855&hgg_type=knownGene&db=mm10&hgsid=500967801_Vakd6YZAzH49xqeBY7x8QEfmp51u
AB335742	chrY:40,534,264-40,534,307	non-coding RNA, oocyte_clustered_small_RNA943, complete sequence	http://genome.ucsc.edu/cgi-bin/hgGene?hgg_gene=uc029xxv.1&hgg_prot=&hgg_chrom=chrY&hgg_start=40534263&hgg_end=40534307&hgg_type=knownGene&db=mm10&hgsid=500967801_Vakd6YZAzH49xqeBY7x8QEfmp51u
AB349653	chrY:18,895,613-18,895,656	non-coding RNA, oocyte_piRNA469, complete sequence	http://genome.ucsc.edu/cgi-bin/hgGene?hgg_gene=uc029xse.1&hgg_prot=&hgg_chrom=chrY&hgg_start=18895612&hgg_end=18895656&hgg_type=knownGene&db=mm10&hgsid=500967801_Vakd6YZAzH49xqeBY7x8QEfmp51u
AB349653	chrY:14,183,345-14,183,388	non-coding RNA, oocyte_piRNA469, complete sequence	http://genome.ucsc.edu/cgi-bin/hgGene?hgg_gene=uc029xsb.1&hgg_prot=&hgg_chrom=chrY&hgg_start=14183344&hgg_end=14183388&hgg_type=knownGene&db=mm10&hgsid=500967801_Vakd6YZAzH49xqeBY7x8QEfmp51u
AB349653	chrY:14,183,345-14,183,388	non-coding RNA, oocyte_piRNA469, complete sequence	http://genome.ucsc.edu/cgi-bin/hgGene?hgg_gene=uc029xsb.1&hgg_prot=&hgg_chrom=chrY&hgg_start=14183344&hgg_end=14183388&hgg_type=knownGene&db=mm10&hgsid=500967801_Vakd6YZAzH49xqeBY7x8QEfmp51u
AK006653	chrY:15,155,239-15,178,893	adult male testis cDNA, RIKEN full-length enriched library, clone:1700040F15	http://genome.ucsc.edu/cgi-bin/hgGene?hgg_gene=uc009vft.1&hgg_prot=&hgg_chrom=chrY&hgg_start=15155238&hgg_end=15178893&hgg_type=knownGene&db=mm10&hgsid=500967801_Vakd6YZAzH49xqeBY7x8QEfmp51u
AK006653	chrY:15,155,239-15,178,893	adult male testis cDNA, RIKEN full-length enriched library, clone:1700040F15	http://genome.ucsc.edu/cgi-bin/hgGene?hgg_gene=uc009vft.1&hgg_prot=&hgg_chrom=chrY&hgg_start=15155238&hgg_end=15178893&hgg_type=knownGene&db=mm10&hgsid=500967801_Vakd6YZAzH49xqeBY7x8QEfmp51u
DQ874391	chrY:45,874,035-45,876,398	amplified spermatogenic transcript Y-encoded	http://genome.ucsc.edu/cgi-bin/hgGene?hgg_gene=uc029xza.1&hgg_prot=&hgg_chrom=chrY&hgg_start=45874034&hgg_end=45876398&hgg_type=knownGene&db=mm10&hgsid=500967801_Vakd6YZAzH49xqeBY7x8QEfmp51u

		mRNA sequence	
DQ874391	chrY:42,856,094-42,858,456	amplified spermatogenic transcript Y-encoded mRNA sequence	http://genome.ucsc.edu/cgi-bin/hgGene?hgg_gene=uc009vfy.1&hgg_prot=&hgg_chrom=chrY&hgg_start=42856093&hgg_end=42858456&hgg_type=knownGene&db=mm10&hgsid=500967801_Vakd6YZAzH49xqeBY7x8QEfmp51u
DQ874391	chrY:31,290,381-31,292,742	amplified spermatogenic transcript Y-encoded mRNA sequence	http://genome.ucsc.edu/cgi-bin/hgGene?hgg_gene=uc029xux.1&hgg_prot=&hgg_chrom=chrY&hgg_start=31290380&hgg_end=31292742&hgg_type=knownGene&db=mm10&hgsid=500967801_Vakd6YZAzH49xqeBY7x8QEfmp51u
DQ874391	chrY:30,423,567-30,425,930	amplified spermatogenic transcript Y-encoded mRNA sequence	http://genome.ucsc.edu/cgi-bin/hgGene?hgg_gene=uc009vhs.1&hgg_prot=&hgg_chrom=chrY&hgg_start=30423566&hgg_end=30425930&hgg_type=knownGene&db=mm10&hgsid=500967801_Vakd6YZAzH49xqeBY7x8QEfmp51u
FJ541075	chrY:46,481,356-46,536,969	piRNA piRY splice variant 1, precursor RNA, partial sequence	http://genome.ucsc.edu/cgi-bin/hgGene?hgg_gene=uc029xzb.1&hgg_prot=&hgg_chrom=chrY&hgg_start=46481355&hgg_end=46536969&hgg_type=knownGene&db=mm10&hgsid=500967801_Vakd6YZAzH49xqeBY7x8QEfmp51u
FJ541078	chrY:19,868,054-19,891,496	piRNA piRY splice variant 4, precursor RNA, partial sequence	http://genome.ucsc.edu/cgi-bin/hgGene?hgg_gene=uc029xsf.1&hgg_prot=&hgg_chrom=chrY&hgg_start=19868053&hgg_end=19891496&hgg_type=knownGene&db=mm10&hgsid=500967801_Vakd6YZAzH49xqeBY7x8QEfmp51u
FJ541087	chrY:20,697,597-20,753,364	piRNA piRY splice variant 13, precursor RNA, partial sequence	http://genome.ucsc.edu/cgi-bin/hgGene?hgg_gene=uc029xsh.1&hgg_prot=&hgg_chrom=chrY&hgg_start=20697596&hgg_end=20753364&hgg_type=knownGene&db=mm10&hgsid=500967801_Vakd6YZAzH49xqeBY7x8QEfmp51u
FJ541095	chrY:43,117,378-43,173,007	piRNA piRY splice variant 21, precursor RNA, partial sequence	http://genome.ucsc.edu/cgi-bin/hgGene?hgg_gene=uc029xyb.1&hgg_prot=&hgg_chrom=chrY&hgg_start=43117377&hgg_end=43173007&hgg_type=knownGene&db=mm10&hgsid=500967801_Vakd6YZAzH49xqeBY7x8QEfmp51u
FJ541095	chrY:42,533,944-42,568,599	piRNA piRY splice variant 21, precursor RNA, partial sequence	http://genome.ucsc.edu/cgi-bin/hgGene?hgg_gene=uc029xxw.1&hgg_prot=A0A087WNW5&hgg_chrom=chrY&hgg_start=42533932&hgg_end=42582060&hgg_type=knownGene&db=mm10&hgsid=500967801_Vakd6YZAzH49xqeBY7x8QEfmp51u

Gm20736	chrY:28,245,548-28,270,080	predicted gene, 20736 (Gm20736), mRNA	http://genome.ucsc.edu/cgi-bin/hgGene?hgg_gene=uc029xte.1&hgg_prot=A0A087WSR0&hgg_chrom=chrY&hgg_start=28245356&hgg_end=28271524&hgg_type=knownGene&db=mm10&hgsid=500967801_Vakd6YZAzH49xqeBY7x8QEfmp51u
Gm20736	chrY:22,835,282-22,859,834	predicted gene, 20736 (Gm20736), mRNA	http://genome.ucsc.edu/cgi-bin/hgGene?hgg_gene=uc029xsp.1&hgg_prot=J3QNV4&hgg_chrom=chrY&hgg_start=22835090&hgg_end=22861268&hgg_type=knownGene&db=mm10&hgsid=500967801_Vakd6YZAzH49xqeBY7x8QEfmp51u
Gm20736	chrY:20,967,673-20,992,230	predicted gene, 20736 (Gm20736), mRNA	http://genome.ucsc.edu/cgi-bin/hgGene?hgg_gene=uc029xsl.1&hgg_prot=A0A087WSR0&hgg_chrom=chrY&hgg_start=20967481&hgg_end=20993669&hgg_type=knownGene&db=mm10&hgsid=500967801_Vakd6YZAzH49xqeBY7x8QEfmp51u
Gm20747	chrY:21,166,083-21,166,766	predicted gene, 20747 (Gm20747), mRNA	http://genome.ucsc.edu/cgi-bin/hgGene?hgg_gene=uc029xsm.1&hgg_prot=Q5FWB5&hgg_chrom=chrY&hgg_start=21164553&hgg_end=21166898&hgg_type=knownGene&db=mm10&hgsid=500967801_Vakd6YZAzH49xqeBY7x8QEfmp51u
Gm20806	chrY:27,692,628-27,693,311	predicted gene, 20806 (Gm20806), mRNA	http://genome.ucsc.edu/cgi-bin/hgGene?hgg_gene=uc029xtd.1&hgg_prot=Q9D9T3&hgg_chrom=chrY&hgg_start=27691135&hgg_end=27693439&hgg_type=knownGene&db=mm10&hgsid=500967801_Vakd6YZAzH49xqeBY7x8QEfmp51u
Gm20806	chrY:26,947,544-26,948,227	predicted gene, 20806 (Gm20806), mRNA	http://genome.ucsc.edu/cgi-bin/hgGene?hgg_gene=uc029xtc.1&hgg_prot=Q9D9T3&hgg_chrom=chrY&hgg_start=26946051&hgg_end=26948355&hgg_type=knownGene&db=mm10&hgsid=500967801_Vakd6YZAzH49xqeBY7x8QEfmp51u
Gm20809	chrY:25,995,821-25,996,504	predicted gene, 20809 (Gm20809), mRNA	http://genome.ucsc.edu/cgi-bin/hgGene?hgg_gene=uc029xta.1&hgg_prot=Q62458&hgg_chrom=chrY&hgg_start=25995643&hgg_end=25996635&hgg_type=knownGene&db=mm10&hgsid=500967801_Vakd6YZAzH49xqeBY7x8QEfmp51u
Gm20809	chrY:25,482,701-25,483,384	predicted gene, 20809 (Gm20809), mRNA	http://genome.ucsc.edu/cgi-bin/hgGene?hgg_gene=uc029xsy.1&hgg_prot=Q62458&hgg_chrom=chrY&hgg_start=25482523&hgg_end=25483515&hgg_type=knownGene&db=mm10&hgsid=500967801_Vakd6YZAzH49xqeBY7x8QEfmp51u
Gm20809	chrY:24,969,606-24,970,289	predicted gene, 20809 (Gm20809), mRNA	http://genome.ucsc.edu/cgi-bin/hgGene?hgg_gene=uc029xsw.1&hgg_prot=Q62458&hgg_chrom=chrY&hgg_start=24969428&hgg_end=24970420&hgg_type=knownGene&db=mm10&hgsid=500967801_Vakd6YZAzH49xqeBY7x8QEfmp51u

Gm20809	chrY:24,456,582-24,457,265	predicted gene, 20809 (Gm20809), mRNA	http://genome.ucsc.edu/cgi-bin/hgGene?hgg_gene=uc029xsu.1&hgg_prot=Q62458&hgg_chrom=chrY&hgg_start=24456404&hgg_end=24457396&hgg_type=knownGene&db=mm10&hgsid=500967801_Vakd6YZAzH49xqeBY7x8QEfmp51u
Gm20809	chrY:23,943,343-23,944,026	predicted gene, 20809 (Gm20809), mRNA	http://genome.ucsc.edu/cgi-bin/hgGene?hgg_gene=uc029xss.1&hgg_prot=Q62458&hgg_chrom=chrY&hgg_start=23943165&hgg_end=23944157&hgg_type=knownGene&db=mm10&hgsid=500967801_Vakd6YZAzH49xqeBY7x8QEfmp51u
Gm20815	chrY:8,832,956-8,833,639	predicted gene, 20815 (Gm20815), mRNA	http://genome.ucsc.edu/cgi-bin/hgGene?hgg_gene=uc012hti.1&hgg_prot=J3KMI0&hgg_chrom=chrY&hgg_start=8832248&hgg_end=8835169&hgg_type=knownGene&db=mm10&hgsid=500967801_Vakd6YZAzH49xqeBY7x8QEfmp51u
Gm20822	chrY:15,882,559-15,883,245	predicted gene, 20822 (Gm20822), mRNA	http://genome.ucsc.edu/cgi-bin/hgGene?hgg_gene=uc012hsg.1&hgg_prot=J3KMI7&hgg_chrom=chrY&hgg_start=15882558&hgg_end=15883245&hgg_type=knownGene&db=mm10&hgsid=500967801_Vakd6YZAzH49xqeBY7x8QEfmp51u
Gm20822	chrY:15,882,559-15,883,245	predicted gene, 20822 (Gm20822), mRNA	http://genome.ucsc.edu/cgi-bin/hgGene?hgg_gene=uc012hsg.1&hgg_prot=J3KMI7&hgg_chrom=chrY&hgg_start=15882558&hgg_end=15883245&hgg_type=knownGene&db=mm10&hgsid=500967801_Vakd6YZAzH49xqeBY7x8QEfmp51u
Gm20822	chrY:11,197,162-11,197,848	predicted gene, 20822 (Gm20822), mRNA	http://genome.ucsc.edu/cgi-bin/hgGene?hgg_gene=uc012hsi.1&hgg_prot=J3KMI7&hgg_chrom=chrY&hgg_start=11197161&hgg_end=11197848&hgg_type=knownGene&db=mm10&hgsid=500967801_Vakd6YZAzH49xqeBY7x8QEfmp51u
Gm20826	chrY:7,167,094-7,167,777	predicted gene, 20826 (Gm20826), mRNA	http://genome.ucsc.edu/cgi-bin/hgGene?hgg_gene=uc012hsn.2&hgg_prot=J3QK38&hgg_chrom=chrY&hgg_start=7167019&hgg_end=7636973&hgg_type=knownGene&db=mm10&hgsid=500967801_Vakd6YZAzH49xqeBY7x8QEfmp51u
Gm20831	chrY:17,966,778-17,967,476	predicted gene, 20831 (Gm20831), mRNA	http://genome.ucsc.edu/cgi-bin/hgGene?hgg_gene=uc009vfw.1&hgg_prot=Q3TTD8&hgg_chrom=chrY&hgg_start=17965159&hgg_end=17967598&hgg_type=knownGene&db=mm10&hgsid=500967801_Vakd6YZAzH49xqeBY7x8QEfmp51u
Gm20831	chrY:17,966,778-17,967,476	predicted gene, 20831 (Gm20831), mRNA	http://genome.ucsc.edu/cgi-bin/hgGene?hgg_gene=uc009vfw.1&hgg_prot=Q3TTD8&hgg_chrom=chrY&hgg_start=17965159&hgg_end=17967598&hgg_type=knownGene&db=mm10&hgsid=500967801_Vakd6YZAzH49xqeBY7x8QEfmp51u

Gm20831	chrY:13,254,009-13,254,707	predicted gene, 20831 (Gm20831), mRNA	http://genome.ucsc.edu/cgi-bin/hgGene?hgg_gene=uc029xrz.1&hgg_prot=Q3TTD8&hgg_chrom=chrY&hgg_start=13252390&hgg_end=13254829&hgg_type=knownGene&db=mm10&hgsid=500967801_Vakd6YZAzH49xqeBY7x8QEfmp51u
Gm20837	chrY:44,209,133-44,263,016	-	http://genome.ucsc.edu/cgi-bin/hgGene?hgg_gene=uc029xyf.1&hgg_prot=A0A087WP97&hgg_chrom=chrY&hgg_start=44209121&hgg_end=44263039&hgg_type=knownGene&db=mm10&hgsid=500967801_Vakd6YZAzH49xqeBY7x8QEfmp51u
Gm20837	chrY:30,682,565-30,737,131	-	http://genome.ucsc.edu/cgi-bin/hgGene?hgg_gene=uc029xuc.1&hgg_prot=A0A087WP97&hgg_chrom=chrY&hgg_start=30682553&hgg_end=30737154&hgg_type=knownGene&db=mm10&hgsid=500967801_Vakd6YZAzH49xqeBY7x8QEfmp51u
Gm20837	chrY:29,628,689-29,683,370	-	http://genome.ucsc.edu/cgi-bin/hgGene?hgg_gene=uc029xtf.1&hgg_prot=A0A087WP97&hgg_chrom=chrY&hgg_start=29628677&hgg_end=29683393&hgg_type=knownGene&db=mm10&hgsid=500967801_Vakd6YZAzH49xqeBY7x8QEfmp51u
Gm20857	chrY:26,241,092-26,267,093	predicted gene, 20857 (Gm20857), non-coding RNA	http://genome.ucsc.edu/cgi-bin/hgGene?hgg_gene=uc029xtb.1&hgg_prot=&hgg_chrom=chrY&hgg_start=26241091&hgg_end=26267093&hgg_type=knownGene&db=mm10&hgsid=500967801_Vakd6YZAzH49xqeBY7x8QEfmp51u
Gm20857	chrY:25,727,936-25,753,940	predicted gene, 20857 (Gm20857), non-coding RNA	http://genome.ucsc.edu/cgi-bin/hgGene?hgg_gene=uc029xsz.1&hgg_prot=&hgg_chrom=chrY&hgg_start=25727935&hgg_end=25753940&hgg_type=knownGene&db=mm10&hgsid=500967801_Vakd6YZAzH49xqeBY7x8QEfmp51u
Gm20857	chrY:25,214,874-25,240,906	predicted gene, 20857 (Gm20857), non-coding RNA	http://genome.ucsc.edu/cgi-bin/hgGene?hgg_gene=uc029xsx.1&hgg_prot=&hgg_chrom=chrY&hgg_start=25214873&hgg_end=25240906&hgg_type=knownGene&db=mm10&hgsid=500967801_Vakd6YZAzH49xqeBY7x8QEfmp51u
Gm20857	chrY:24,701,804-24,727,810	predicted gene, 20857 (Gm20857), non-coding RNA	http://genome.ucsc.edu/cgi-bin/hgGene?hgg_gene=uc029xsv.1&hgg_prot=&hgg_chrom=chrY&hgg_start=24701803&hgg_end=24727810&hgg_type=knownGene&db=mm10&hgsid=500967801_Vakd6YZAzH49xqeBY7x8QEfmp51u
Gm20857	chrY:24,188,659-24,214,668	predicted gene, 20857 (Gm20857), non-coding RNA	http://genome.ucsc.edu/cgi-bin/hgGene?hgg_gene=uc029xst.1&hgg_prot=&hgg_chrom=chrY&hgg_start=24188658&hgg_end=24214668&hgg_type=knownGene&db=mm10&hgsid=500967801_Vakd6YZAzH49xqeBY7x8QEfmp51u

Gm20865	chrY:21,243,092-21,243,775	predicted gene, 20865 (Gm20865), mRNA	http://genome.ucsc.edu/cgi-bin/hgGene?hgg_gene=uc029xsn.1&hgg_prot=Q62460&hgg_chrom=chrY&hgg_start=21242965&hgg_end=21243823&hgg_type=knownGene&db=mm10&hgsid=500967801_Vakd6YZAzH49xqeBY7x8QEfmp51u
Gm20871	chrY:42,930,323-43,978,241	predicted gene, 20871 (Gm20871), non-coding RNA	http://genome.ucsc.edu/cgi-bin/hgGene?hgg_gene=uc029xya.1&hgg_prot=&hgg_chrom=chrY&hgg_start=42930322&hgg_end=43978241&hgg_type=knownGene&db=mm10&hgsid=500967801_Vakd6YZAzH49xqeBY7x8QEfmp51u
Gm20871	chrY:30,499,166-30,523,158	predicted gene, 20871 (Gm20871), non-coding RNA	http://genome.ucsc.edu/cgi-bin/hgGene?hgg_gene=uc029xub.1&hgg_prot=A0A087WSR0&hgg_chrom=chrY&hgg_start=30497728&hgg_end=30523759&hgg_type=knownGene&db=mm10&hgsid=500967801_Vakd6YZAzH49xqeBY7x8QEfmp51u
Gm20877	chrY:17,095,074-17,095,757	predicted gene, 20877 (Gm20877), mRNA	http://genome.ucsc.edu/cgi-bin/hgGene?hgg_gene=uc029xsc.1&hgg_prot=J3QPZ4&hgg_chrom=chrY&hgg_start=17093878&hgg_end=17095887&hgg_type=knownGene&db=mm10&hgsid=500967801_Vakd6YZAzH49xqeBY7x8QEfmp51u
Gm20877	chrY:17,095,074-17,095,757	predicted gene, 20877 (Gm20877), mRNA	http://genome.ucsc.edu/cgi-bin/hgGene?hgg_gene=uc029xsc.1&hgg_prot=J3QPZ4&hgg_chrom=chrY&hgg_start=17093878&hgg_end=17095887&hgg_type=knownGene&db=mm10&hgsid=500967801_Vakd6YZAzH49xqeBY7x8QEfmp51u
Gm20877	chrY:12,383,184-12,383,867	predicted gene, 20877 (Gm20877), mRNA	http://genome.ucsc.edu/cgi-bin/hgGene?hgg_gene=uc012hsk.1&hgg_prot=J3QPZ4&hgg_chrom=chrY&hgg_start=12381988&hgg_end=12383997&hgg_type=knownGene&db=mm10&hgsid=500967801_Vakd6YZAzH49xqeBY7x8QEfmp51u
Gm20917	chrY:23,031,235-23,031,918	predicted gene, 20917 (Gm20917), mRNA	http://genome.ucsc.edu/cgi-bin/hgGene?hgg_gene=uc029xsr.1&hgg_prot=Q62457&hgg_chrom=chrY&hgg_start=23031058&hgg_end=23032050&hgg_type=knownGene&db=mm10&hgsid=500967801_Vakd6YZAzH49xqeBY7x8QEfmp51u
Gm21746	chrY:6,237,453-6,237,995	predicted gene, 21746 (Gm21746), mRNA	https://genome.ucsc.edu/cgi-bin/hgc?hgsid=512004667_nnUUiRL0XAElvOvMqv8RWMa25fHI&c=chrY&l=6119999&r=6570000&o=6237452&t=6237995&g=ensGene&i=ENSMUST00000177612
Gm20873	chrY:6,268,725-6,269,423	predicted gene, 20873 (Gm20873), mRNA	https://genome.ucsc.edu/cgi-bin/hgc?hgsid=512004667_nnUUiRL0XAElvOvMqv8RWMa25fHI&c=chrY&l=6119999&r=6570000&o=6268724&t=6269423&g=ensGene&i=ENSMUST00000179410

AB351778	chrY:6,530,295-6,530,316	Mus musculus non-coding RNA, oocyte_piRNA 2594	https://genome.ucsc.edu/cgi-bin/hgc?hgsid=512004667_nnUUiRL0XAElvOvMqv8RWMa25fHI&c=chrY&l=6119999&r=6570000&o=6530294&t=6530316&g=mrna&i=AB351778
AB349505	chrY:6,530,296-6,530,319	Mus musculus non-coding RNA, oocyte_piRNA 321	https://genome.ucsc.edu/cgi-bin/hgc?hgsid=512004667_nnUUiRL0XAElvOvMqv8RWMa25fHI&c=chrY&l=6119999&r=6570000&o=6530295&t=6530319&g=mrna&i=AB349505
AB349959	chrY:6,530,337-6,530,361	Mus musculus non-coding RNA, oocyte_piRNA 775	https://genome.ucsc.edu/cgi-bin/hgc?hgsid=512004667_nnUUiRL0XAElvOvMqv8RWMa25fHI&c=chrY&l=6119999&r=6570000&o=6530336&t=6530361&g=mrna&i=AB349959
DQ874391	chrY:46,847,641-46,850,004	Mus musculus amplified spermatogenic transcript Y-encoded mRNA sequence	https://genome.ucsc.edu/cgi-bin/hgGene?hgg_gene=uc029xzc.1&hgg_prot=&hgg_chrom=chrY&hgg_start=46847640&hgg_end=46850004&hgg_type=knownGene&db=mm10&hgsid=512004667_nnUUiRL0XAElvOvMqv8RWMa25fHI
Gm21247	chrY:46,757,399-46,758,082	predicted gene, 21247 (Gm21247), mRNA	https://genome.ucsc.edu/cgi-bin/hgc?hgsid=512004667_nnUUiRL0XAElvOvMqv8RWMa25fHI&c=chrY&l=46729999&r=47310000&o=46757398&t=46758082&g=ensGene&i=ENSMUST00000179950
DQ874392	chrY:46,847641-46850004	Mus musculus amplified spermatogenic transcript Y-encoded splice variant mRNA sequence	https://genome.ucsc.edu/cgi-bin/hgc?hgsid=512004667_nnUUiRL0XAElvOvMqv8RWMa25fHI&c=chrY&l=46729999&r=47310000&o=46847640&t=46850004&g=mrna&i=DQ874392
AB340532	chrY:46,826,544-46,826,564	Mus musculus non-coding RNA, oocyte_clustered_small_RNA5733, complete sequence	https://genome.ucsc.edu/cgi-bin/hgc?hgsid=512004667_nnUUiRL0XAElvOvMqv8RWMa25fHI&c=chrY&l=46729999&r=47310000&o=46826543&t=46826564&g=mrna&i=AB340532
FJ386436	chrY:46,872,414-46,872,940	Y-linked testis-specific protein variant 2	https://genome.ucsc.edu/cgi-bin/hgc?hgsid=512004667_nnUUiRL0XAElvOvMqv8RWMa25fHI&c=chrY&l=46729999&r=47310000&o=46872413&t=46872940&g=mrna&i=FJ386436

FJ386435	chrY:46,872,457-46,872,940	Y-linked testis-specific protein variant 1	https://genome.ucsc.edu/cgi-bin/hgc?hgsid=512004667_nnUUiRL0XAElvOvMqv8RWMa25fHI&c=chrY&l=46729999&r=47310000&o=46872456&t=46872940&g=mrna&i=FJ386435
AB349959	chrY:47,156,745-47,156,769	Mus musculus non-coding RNA, oocyte_piRNA 775	https://genome.ucsc.edu/cgi-bin/hgc?hgsid=512004667_nnUUiRL0XAElvOvMqv8RWMa25fHI&c=chrY&l=46729999&r=47310000&o=47156744&t=47156769&g=mrna&i=AB349959
FJ386435	chrY:47,291,729-47,292,212	Mus musculus strain C57BL/6J Y-linked testis-specific protein variant 1 mRNA, partial cds, alternatively spliced	https://genome.ucsc.edu/cgi-bin/hgc?hgsid=512004667_nnUUiRL0XAElvOvMqv8RWMa25fHI&c=chrY&l=46729999&r=47310000&o=47291728&t=47292212&g=mrna&i=FJ386435
Srsy	chrY:17,935,719-17,937,324	serine-rich secreted Y-linked protein	https://genome.ucsc.edu/cgi-bin/hgc?hgsid=512120325_Z7CxwoNZvwGKxJeSPJVIcYKuSQLa&c=chrY&l=17935718&r=17937324&o=17935712&t=17937330&g=mrna&i=EU052291

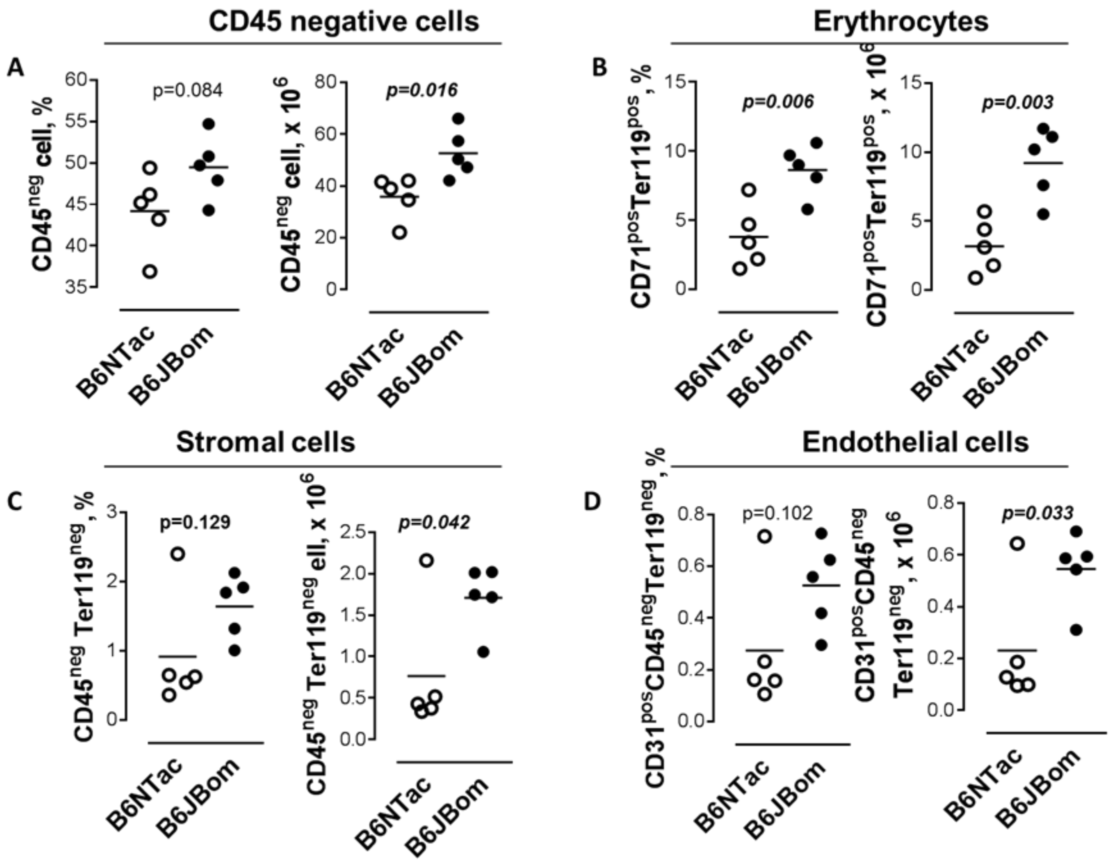

Fig S1. Characterization of changes within CD45^{neg} non-immune subpopulations of B6JBom and B6NTac spleen cells **A)** Percentage and absolute values of total CD45^{neg} population **B)** Percentage and absolute values of erythrocyte subpopulation **C)** Percentage and absolute values of the stromal cell subpopulation **D)** Percentage and absolute values of endothelial cell subpopulation.

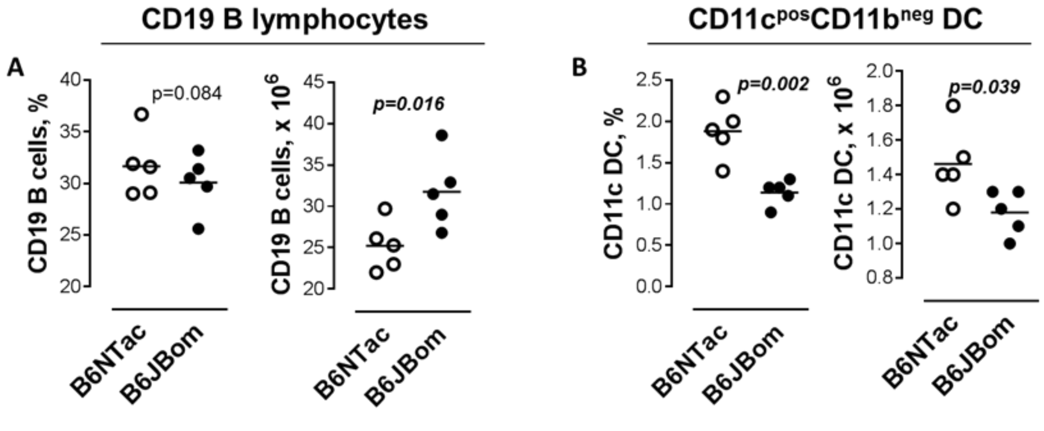

Fig S2. Characterization of changes within CD45^{pos} immune subpopulations of B6JBom and B6NTac spleen cells **A)** Percentage and absolute values of CD19 B lymphocytes **B)** Percentage and absolute values of CD11c^{pos} CD11b^{neg} dendritic cells.

Table S5. Subpopulations of CD45 non-immune and CD45 immune cells in male mouse spleen of B6NTac and B6JBom

% Cell Population	B6NTac			B6JBom			*P* value
	Mean	SEM	n	Mean	SEM	n	
Spleen weight, mg	71.20	2.97	5	88.96	4.91	5	***0.015***
Viable cells, %	90.64	0.57	5	90.50	0.50	5	0.823
$CD45^{neg}$ cells, %	44.18	2.08	5	49.48	1.71	5	0.084
Erythrocytes (CD45-, CD71-, Ter119+), %	39.38	1.18	5	39.12	1.05	5	0.873
Mature erythrocytes (CD45-, CD71+, Ter119+),%	3.80	1.01	5	8.64	0.82	5	***0.006***
Stromal cells (CD45-, CD71-, Ter119-) %	0.92	0.37	5	1.64	0.21	5	0.129
Endothelial cells (CD45-,CD71-, Ter119-, CD31+, Sca1-),%	0.27	0.11	5	0.53	0.08	5	0.102
Fibroblasts (CD45-, CD71-, Ter119-, CD31+, Sca1+), %	0.17	0.15	5	0.36	0.05	5	0.269
Mesenchymal Stem cells (CD45-, CD71-, Ter119-, CD31-, Sca1+),%	0.11	0.04	5	0.18	0.03	5	0.158
$CD45^{pos}$ immune cells, %	55.44	2.06	5	50.06	1.69	5	0.078
T cells (CD45+, CD3+), %	11.06	0.52	5	8.50	0.36	5	***0.004***
T helper cells (CD45+, CD3+, CD4+), %	6.60	0.38	5	5.30	0.35	5	***0.037***
Cytotoxic T cells (CD45+, CD3+, CD8+), %	3.34	0.16	5	2.24	0.15	5	***0.001***
B lymphocytes (CD45+, CD19+), %	31.66	1.40	5	30.08	1.26	5	0.425
Natural killer cells (CD45+, NK1.1+), %	3.50	0.14	5	2.46	0.18	5	***0.002***
Myeloid cells (CD45+, CD11b+, CD11c+, %	2.54	0.11	5	2.30	0.27	5	0.433
Myeloid phagocytic cells (CD45+, CD11c+,MHCII/F4/80+), %	1.88	0.15	5	1.14	0.07	5	***0.002***
Neutrophils (CD45+, CD11b+, Gr-1+), %	0.90	0.12	5	0.87	0.18	5	0.878
Macrophages (CD45+, CD11b+, MHCII/F4/80+, GR1-), %	0.41	0.05	5	0.23	0.04	5	***0.018***
Total Number of Cells							
Total cell, x 10^6	80.40	5.78	5	106.00	6.40	5	***0.018***
CD45-cells, x 10^6	35.86	3.68	5	52.60	4.17	5	***0.016***
Erythrocytes (CD45-, CD71-, Ter119+) erythrocytes, x 10^6	31.88	2.91	5	41.56	3.12	5	0.053
Mature erythrocytes (CD45-, CD71+,Ter119+), x 10^6	3.18	0.86	5	9.22	1.16	5	***0.003***
Stromal cells (CD45-, CD71-, Ter119-), x 10^6	0.76	0.35	5	1.71	0.18	5	***0.042***
Endothelial cells (CD45-, CD71-, Ter119-, CD31+, Sca1-), x 10^6	0.23	0.10	5	0.55	0.06	5	***0.033***
Fibroblasts (CD45-, CD71-, Ter119-, CD31+, Sca1+), x 10^6	0.15	0.14	5	0.37	0.04	5	0.158

Mesenchymal stem cells (CD45-, CD71-, Ter119-, CD31-, Sca1+), x 10^{6}	0.09	0.04	5	0.19	0.02	5	0.051
CD45^{pos} immune cells, x 10^{6}	44.24	2.44	5	52.90	2.95	5	0.053
T cells (CD45+,CD3+), x 10^{6}	8.82	0.65	5	8.92	0.36	5	0.896
T helper cells (CD45+, CD3+, CD4+), x 10^{6}	5.26	0.41	5	5.56	0.31	5	0.578
Cytotoxic T cells (CD45+, CD3+, CD8+), x 10^{6}	2.68	0.19	5	2.34	0.14	5	0.185
B cells (CD45+, CD19+), x 10^{6}	25.22	1.34	5	31.76	2.00	5	***0.027***
Natural killer cells (CD45+, NK1.1+), x 10^{6}	2.82	0.22	5	2.62	0.23	5	0.544
Myeloid cells (CD45+, CD11b+, CD11c+, x 10^{6}	2.04	0.16	5	2.40	0.26	5	0.268
Myeloid phagocytic cells (CD45+, CD11c+, MHCII/F4/80)+, x 10^{6}	1.46	0.10	5	1.18	0.06	5	***0.039***
Neutrophils (CD45+, Gr-1+,CD11b+), x 10^{6}	0.74	0.12	5	0.92	0.18	5	0.421
Macrophages (CD45+, F4/80+, Gr-1-, CD11b+, x 10^{6}	0.30	0.03	5	0.24	0.05	5	0.346

Table S6. Immune-cell subpopulations of male mouse bone marrow in B6NTac and B6JBom

	**B6NTac**			**B6JBom**			*P* value‡
**% Cell Population**	Mean	SEM	N	Mean	SEM	N	
Viable cells, %	90.6	0.4	5	90.5	0.4	5	0.807
CD45^{pos} immune cells, %	54.8	2.3	5	48.4	2.9	5	0.117
CD11b^{pos} myeloid cells, %	35.0	2.6	5	29.1	2.0	5	0.119
Ly6G^{pos}Ly6C^{low}CD11b^{pos} granulocytes, %	23.2	1.9	5	18.9	1.5	5	0.113
Ly6G^{neg}Ly6C^{neg}CD11b^{pos} monocytes, %	10.7	0.9	5	8.9	0.6	5	0.126
F4/80^{pos}CD11b^{pos}Ly6G^{neg} macrophages&APC, %	0.86	0.1	5	0.88	0.1	5	0.852
CD19^{pos} B lymphocytes, %	11.0	0.3	5	11.0	0.5	5	0.973
lin^{neg}CD45^{pos} hematopoietic stem/progenitor cells,* %	2.2	0.1	5	2.0	0.1	5	0.176
Total number of viable cells,† x 10^{6}	48.2	3.7	5	45.2	3.6	5	0.579
CD45^{pos} immune cells, x 10^{6}	26.4	2.1	5	21.5	1.3	5	0.087
CD11b^{pos} myeloid cells, x 10^{6}	16.7	1.7	5	12.9	0.8	5	0.076
Ly6G^{pos}Ly6C^{low}CD11b^{pos} granulocytes, x 10^{6}	11.1	1.1	5	8.4	0.5	5	0.051
Ly6G^{neg}Ly6C^{neg}CD11b^{pos} monocytes, x 10^{6}	5.2	0.6	5	4.0	0.3	5	0.12
F4/80^{po}sCD11b^{pos}Ly6G^{neg} macrophages&APC, x 10^{6}	0.41	0.0	5	0.4	0.0	5	0.885
CD19^{pos} B lymphocytes, x 10^{6}	5.3	0.5	5	4.9	0.3	5	0.518
lin^{neg}CD45^{pos} hematopoietic stem/progenitor cells, x 10^{6}	1.1	0.1	5	0.9	0.1	5	0.115

* - percent of HPC was found within CD45^{pos} cells after gating out CD11b, CD19, Ly6G, Ly6C and F4/80 positive populations.

† - total number of cells from four bones (left and right legs)
‡ - unpaired *t* test
